# Five-Year Evaluation of the PROA-NEN Pediatric Antimicrobial Stewardship Program in a Spanish Tertiary Hospital

**DOI:** 10.3390/antibiotics13060511

**Published:** 2024-05-30

**Authors:** Aurora Fernández-Polo, Susana Melendo-Perez, Nieves Larrosa Escartin, Natalia Mendoza-Palomar, Marie Antoinette Frick, Pere Soler-Palacin, on behalf of the PROA-NEN Working Group

**Affiliations:** 1 Pharmacy Department, Hospital Infantil, Institut de Recerca Vall d’Hebron, Vall d’Hebron Barcelona Hospital Campus, 08035 Barcelona, Spain; 2 Pediatric Infectious Diseases and Immunodeficiencies Unit, Hospital Infantil, Institut de Recerca Vall d’Hebron, Vall d’Hebron Barcelona Hospital Campus, 08035 Barcelona, Spain; susana.melendo@vallhebron.cat (S.M.-P.); nataliaana.mendoza@vallhebron.cat (N.M.-P.); marieantoinette.frick@vallhebron.cat (M.A.F.); pere.soler@vallhebron.cat (P.S.-P.); 3 Microbiology Department, Institut de Recerca Vall d’Hebron, Universitat Autònoma de Barcelona, Vall d’Hebron Barcelona Hospital Campus, 08035 Barcelona, Spain; nieves.larrosa@vallhebron.cat; 4 CIBER de Enfermedades Infecciosas (CIBERINFEC), Instituto de Salud Carlos III, 28029 Madrid, Spain

**Keywords:** antimicrobial stewardship, drug resistance, bacterial infections, mycoses, tertiary care centers, pharmaceutical economics

## Abstract

**Introduction:** Actions to reduce and optimize antimicrobial use are crucial in the management of infectious diseases to counteract the emergence of short- and long-term resistance. This is particularly important for pediatric patients due to the increasing incidence of serious infections caused by resistant bacteria in this population. The aim of this study was to evaluate the impact of a pediatric antimicrobial stewardship program (PROA-NEN) implemented in a Spanish tertiary hospital by assessing the use of systemic antimicrobials, clinical indicators, antimicrobial resistance, and costs. **Methods:** In this quasi-experimental, single-center study, we included pediatric patients (0–18 years) admitted to specialized pediatric medical and surgical units, as well as pediatric and neonatal intensive care units, from January 2015 to December 2019. The impact of the PROA-NEN program was assessed using process (consumption trends and prescription quality) and outcome indicators (clinical and microbiological). Antibiotic prescription quality was determined using quarterly point prevalence cross-sectional analyses. **Results:** Total antimicrobial consumption decreased during the initial three years of the PROA-NEN program, followed by a slight rebound in 2019. This decrease was particularly evident in intensive care and surgical units. Antibiotic use, according to the WHO Access, Watch and Reserve (AWaRe) classification, remained stable during the study period. The overall rate of appropriate prescription was 83.2%, with a significant increase over the study period. Clinical indicators did not substantially change over the study period. Direct antimicrobial expenses decreased by 27.3% from 2015 to 2019. **Conclusions:** The PROA-NEN program was associated with reduced antimicrobial consumption, improved appropriate use, and decreased costs without compromising clinical and/or microbiological outcomes in patients.

## 1. Introduction

The use of antimicrobial agents to treat infections inevitably leads to the emergence of antimicrobial resistance [1]. This is particularly concerning in the hospital setting due to the extensive use of antimicrobials combined with the vulnerability of hospitalized patients.

To address this challenge, efforts to reduce and optimize antimicrobial use at all levels of healthcare are vital. This includes global initiatives like the Global Action Plan on Antimicrobial Resistance [2] and country-specific strategies such as the Spanish *Plan Nacional frente a la Resistencia a los Antibióticos* (PRAN) [3]. In this context, antimicrobial stewardship programs (ASPs) are a comprehensive set of coordinated, long-term actions and interventions within health institutions to promote the optimal use of antimicrobials and ensure sustainable access to antimicrobial therapy [4,5,6,7,8,9,10].

In the pediatric population, recent years have seen a rising incidence of serious infections caused by resistant bacteria, leading to increased morbidity and mortality [11,12,13,14,15]. Furthermore, the prevalence of antibiotic resistance is different in pediatric and adult hospitals [16]. That is, because pediatric patients differ significantly from adults in terms of clinical presentation, progression of infections, existing comorbidities, and mortality rates, antimicrobial treatments and ASPs should be tailored to this population.

The *Programa de Optimización de Antimicrobiano* (PROA-NEN) is a non-restrictive pediatric-specific ASP established in 2015 at the Children’s Hospital at Vall d’Hebron Barcelona Hospital Campus (Barcelona, Spain). The program was implemented to improve the use of antimicrobial treatments for pediatric patients admitted to the hospital by reducing their consumption, increasing their efficiency, and improving clinical and ecological outcomes. The objective of this study was to evaluate the effects of the PROA-NEN program on the use of systemic antimicrobials, clinical indicators, and antimicrobial resistance rates, and its economic impact within a public health system where sustainability is crucial.

## 2. Results

### 2.1. Antibiotic Prescription Quality

A total of 1013 antibiotic prescriptions were evaluated in 652 patients. Overall, 61.1% were prescribed in specialist medical units, 16.7% in the pediatric intensive care unit (P-ICU), 15.1% in the neonatal intensive care unit (N-ICU), and 7.1% in surgical units. Half (50.5%) of the prescriptions were administered as empirical treatment, 18.9% as targeted treatment, and 30.6% as prophylactic treatment.

**Figure 1:**
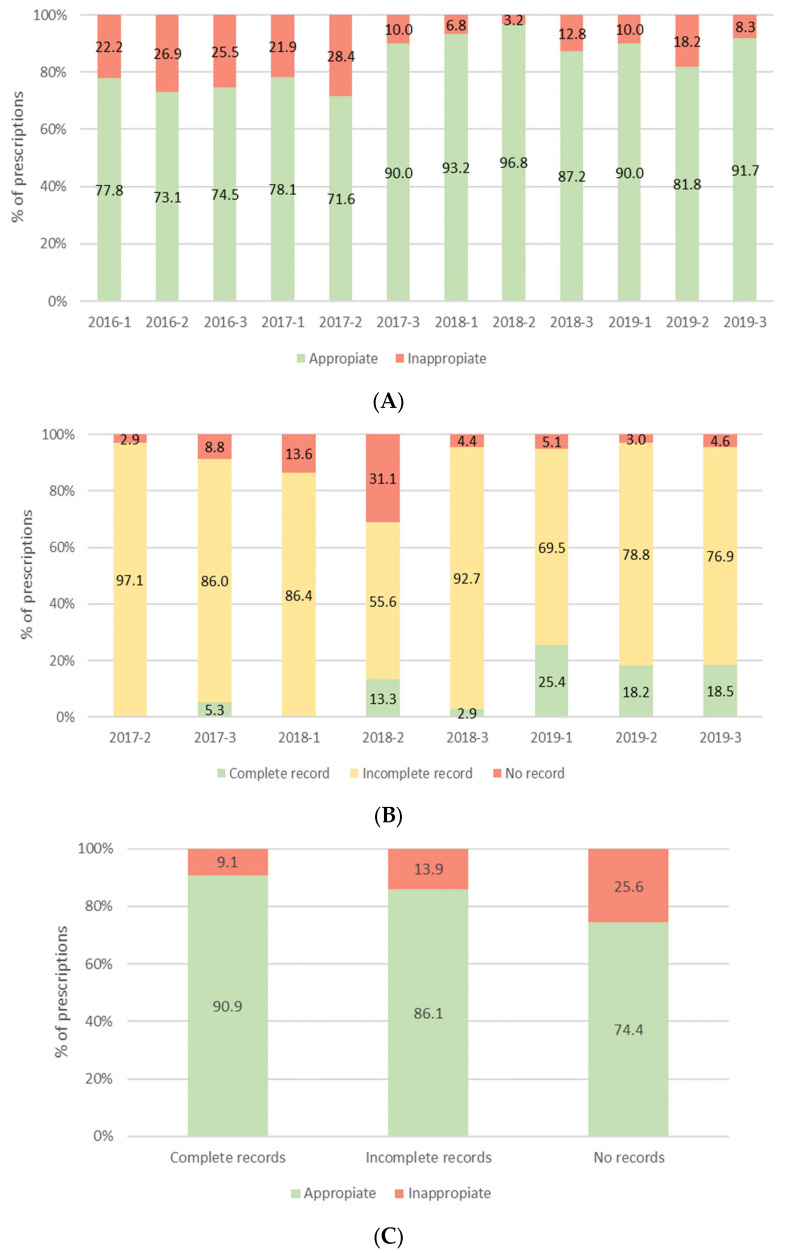
Antibiotic prescription quality in cross-sectional analyses. Stacked bars show (**A**) the proportion of appropriate and inappropriate prescription rates, (**B**) the proportion of complete, incomplete, or no records, and (**C**) the proportion of appropriate and inappropriate prescribing according to record completeness (complete, incomplete, no records).

Of the total antibiotic prescriptions, 843 were appropriate, representing a prescription appropriateness rate of 83.2%. As seen in Figure 1A, the rate of appropriate prescription was >70% in the first five cross-sectional analyses and significantly improved throughout the study period (*p* < 0.0001). As of the fifth cross-sectional analysis, the distribution of antibiotic prescription record completeness was determined. A total of 455 prescriptions from 321 patients were analyzed, of which 47 (10.5%) had a complete record. The percentage of complete records improved over the study period analyzed (Figure 1B). The highest proportion of appropriate prescriptions was observed for treatments with complete records, followed by those with incomplete records, while treatments with no records showed the lowest rate of appropriate prescriptions (Figure 1C). The percentage of appropriate prescriptions during the study period was 75.2% in the P-ICU, 79.2% in surgical units, 82.8% in specialist medical units, and 82.4% in the N-ICU.

### 2.2. Antibiotic Consumption according to the AWaRe Classification

**Figure 2:**
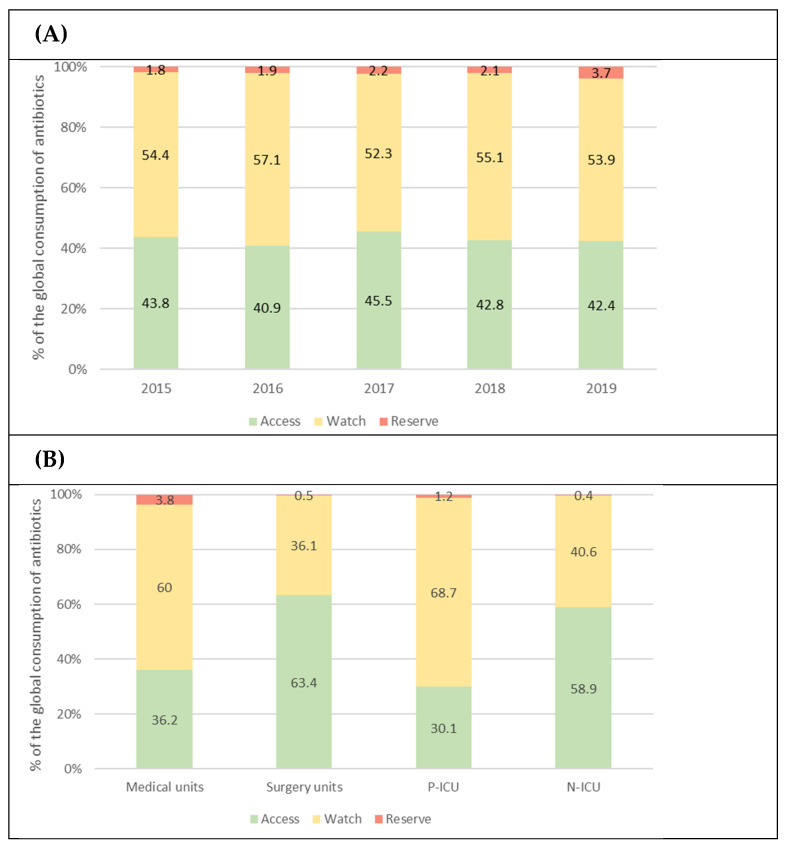
Proportion of WHO AWaRe antimicrobial consumption. Stacked bars show the proportion of Access, Watch and Reserved antibiotics consumed (**A**) in each year of the study, and (**B**) across clinical units. N-ICU, neonatal intensive care unit; P-ICU, pediatric intensive care unit.

Antibiotic use, according to the WHO Essential Medicines List Access, Watch, and Reserve (AWaRe) classification, did not show substantial changes over the study period. Access antibiotics accounted for 43.1% of the total consumption, Watch antibiotics represented 54.6%, and Reserve antibiotics ranged from 1.8% to 2.2% between 2015 and 2018 and reached 3.7% in 2019 (Figure 2A).

The use of Access antibiotics was higher in surgical units (63.4% of all antibiotics consumed) and the N-ICU (58.9%). The highest percentage of Reserve antibiotics was observed in specialist medical units, accounting for up to 3.8% of the total antibiotics used (Figure 2B).

### 2.3. Antimicrobial Consumption Trends

**Figure 3:**
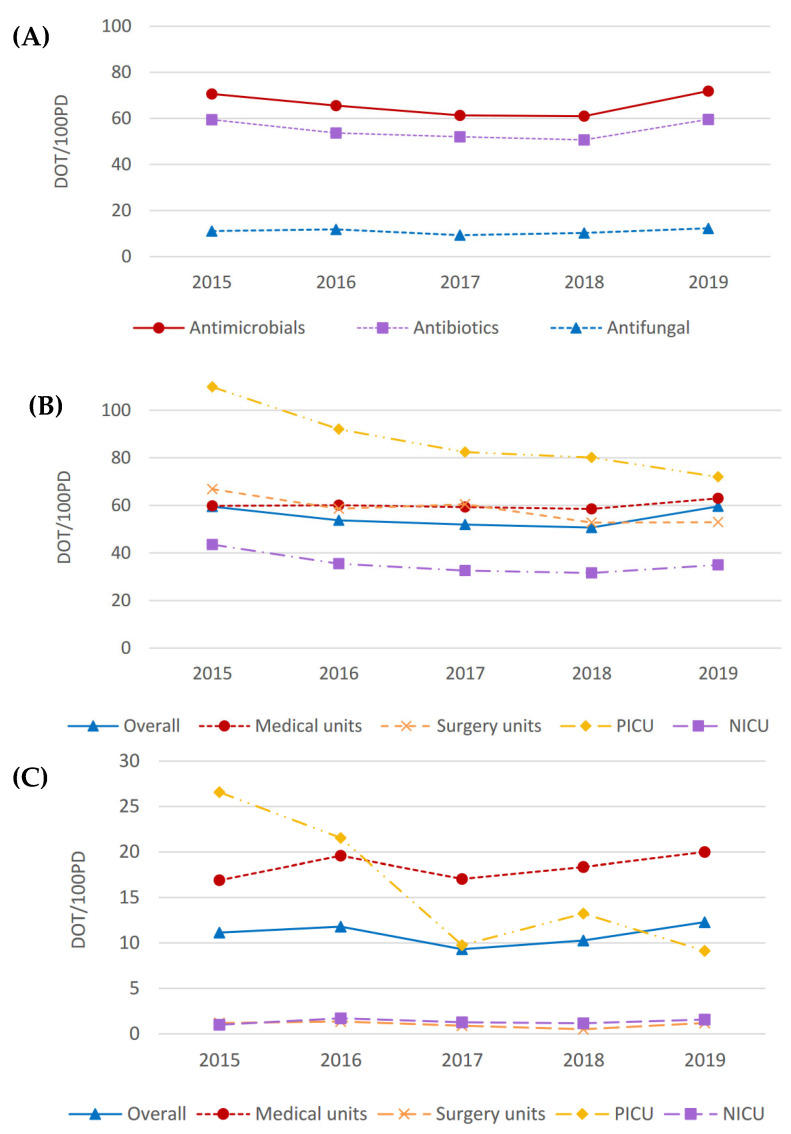
Antimicrobial consumption over the study period. The graphs show (**A**) total antimicrobial, antibiotic, and antifungal consumption over the study period, (**B**) antibiotic consumption across clinical units, and (**C**) antifungal consumption across clinical units. Data are expressed as DOT/100PD. N-ICU, neonatal intensive care unit; P-ICU, pediatric intensive care unit.

The most commonly used antibiotics throughout the study period are shown in Table 1, expressed as days of therapy (DOT)/100 patient days (PD). Total antimicrobial consumption was 70.6 DOT/100 PD in 2015, followed by a decrease within the first three years after introducing the program and an increase in 2019 (71.9 DOT/100 PD). Antibiotic use followed the same pattern, a consumption of 59.5 DOT/100 PD in 2015 that decreased to 50.7 DOT/100 PD in 2018 and increased in 2019 (59.6 DOT/100PD). Antifungal use was around five times lower than antibiotic use and remained stable throughout the study period (Figure 3A). There were no statistically significant differences in the consumption of antimicrobials (*p* = 0.92), antibiotics (*p* = 0.86), or antifungals (*p* = 0.87) throughout the years.

**Figure 4:**
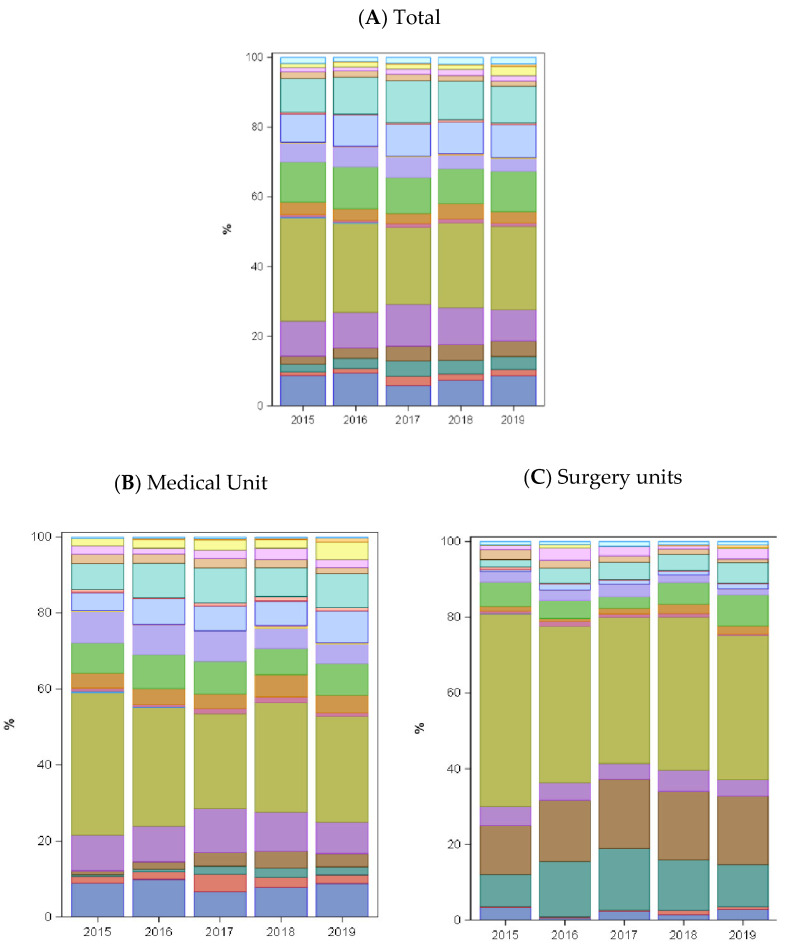

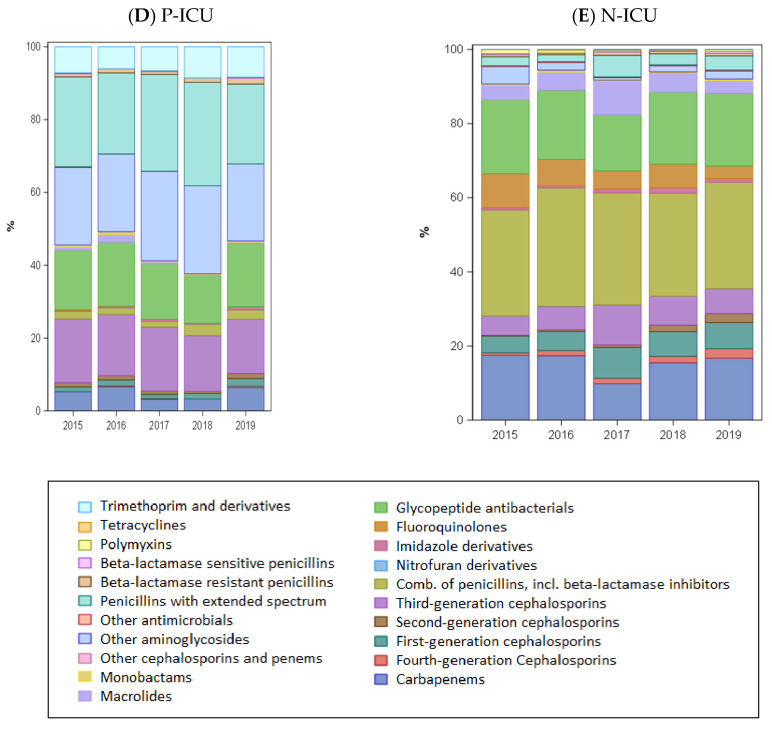
Proportion of antibiotics consumed over the study period. Stacked bars represent the annual proportion of each antibiotic class, both overall and by clinical unit. Antibiotic consumption is expressed as % DOT/100 PD and categorized using the ATC classification. N-ICU, neonatal intensive care unit; P-ICU, pediatric intensive care unit.

The highest antibiotic use was recorded in the P-ICU, whereas the N-ICU showed the lowest antibiotic consumption. There was a significant decrease in antibiotic use over the study period in the P-ICU (mean ± standard deviation [SD], −8.76 ± 1.56; *p* < 0.05) and surgical units (mean ± SD, −3.36 ± 0.89; *p* < 0.05). The consumption trend in specialist medical units remained stable, with a slight increase in 2019 (Figure 3B). Regarding antifungal agents, P-ICUs showed a significant decrease in their consumption (mean ± SD, −4.31 ± 1.28; *p* < 0.05), while the consumption in other units remained stable (Figure 3C). Figure 4 shows changes in the percentage of antibiotics consumed throughout the study period and across clinical units. We observed that the spectrum of antibiotics used was narrowed over the study period. This was evidenced in surgical units, where the proportion of penicillin with beta-lactamase inhibitors decreased in favor of increased use of 1st- and 2nd-generation cephalosporins. A summary of studies evaluating antimicrobial consumption after the implementation of an ASP is shown in the Supplementary Materials.

### 2.4. Clinical and Resistance Indicators

Clinical indicators did not substantially change over the study period, with a slight decrease in the length of stay (LOS), annual readmission rates, and infection-related mortality rates (Table 2). Infection-related mortality rates decreased from the start to the end of the study period (13.0% vs. 27.6%); however, the differences were not statistically significant (*p* = 0.484).

Changes in the indicators selected for monitoring antimicrobial resistance to the most relevant nosocomial pathogens are detailed in Table 3. We observed a higher prevalence of amoxicillin-clavulanic acid-resistant *Escherichia coli* over the study period due to a change in the analytical method and reference standards. We also observed a decrease in extended-spectrum beta-lactamase-producing (ESBL) *Klebsiella pneumonia*, *Enterobacter cloacae* strains with chromosomal AmpC hyperproduction, and meropenem-resistant *Pseudomonas aeruginosa*. There were no significant differences in the percentage of ESBL *Escherichia coli* and extensively drug-resistant (XDR) *Pseudomonas aeruginosa* according to the criteria of Magiorakos et al. [17]. Similarly, for Gram-positive strains, there were no significant differences in the percentage of methicillin-resistant *Staphylococcus aureus*. No patients with multidrug-resistant *Acinetobacter baumannii* or vancomycin-resistant enterococci were identified during the study period.

### 2.5. Complexity of Care

The mean ± SD number of annual stays was 59,523 ± 2514, with no substantial variations during the study period. The complexity of care, measured by the weighted mean of the diagnosis-related groups (WM-DRG), increased over the study period: 1.32 in 2015, 1.40 in 2016, 1.36 in 2017, 1.38 in 2018, 1.49 in 2019, and 1.61 in 2020. However, no linear relationship was observed between WM-DRG and DOT/100 PD (r = 0.35), indicating that there was no correlation between the increase in WM-DRG and the total consumption of antimicrobials.

### 2.6. Costs

The centralized preparation of systemic antimicrobials for intravenous administration in pharmacy departments offered savings of 30–35% in annual antimicrobial expenditure, resulting in cumulative savings of €1,210,376 (mean annual savings of €242,000) between 2015 and 2019. The number of preparations over the study period was as follows: 5064 in 2015, 4812 in 2016, 3408 in 2017, 4547 in 2018, and 5280 in 2019.

## 3. Discussion

This study identified a declining trend in antimicrobial consumption during the first three years following the implementation of the PROA-NEN program, followed by a rebound in 2019 that disrupted this trend. Antimicrobial consumption rates were lower than those reported in recent studies conducted in tertiary pediatric hospitals in different settings [18,19,20,21]. The use of Reserve antibiotics was limited and their prescription in neonates and critical and surgical units was minimal. An increase in appropriate prescriptions was also observed during the study period.

Pediatric ASPs require tailored approaches due to the unique clinical characteristics of the pediatric population. This includes the need for validated measurement units [22,23]. Numerous scientific societies and healthcare stakeholders have underscored the importance of prioritizing ASP development in pediatrics and have set forth specific recommendations for this purpose [23,24,25,26,27,28,29,30,31]. In our setting, the VINCat pediatric PROA Sharing Antimicrobial Reports for Pediatric Stewardship (SHARP) survey was the first overview of the current state of pediatric antimicrobial stewardship programs in hospitals across Catalonia. This survey revealed that while most hospitals have implemented measures suggested by pediatric ASPs, many have not established their own dedicated program. It is crucial to provide these programs with more resources so that these hospitals can create tailored initiatives and define specific indicators for this age group [32].

A national consensus document emphasized the need for antimicrobial protocols tailored specifically to children, instead of merely adapting adult ones with dosage adjustments [33]. However, the scientific literature on the development of ASPs in pediatric and neonatal populations is still much more limited than in the adult population [33,34], with wide variability in methodology, program durations, and population scope [35]. In this context, this is the first study to comprehensively evaluate consumption trends, prescription quality, clinical and microbiological outcomes, and costs of a pediatric ASP. Unlike previous studies, we evaluated the efficiency of the program by including all pediatric patients admitted to our center instead of focusing on a single area or department. It is important to note that we excluded data from 2020 due to the extraordinary disruptions caused by the COVID-19 pandemic.

In 2019, we observed an increase in antimicrobial consumption, which may be attributed to several significant developments that occurred within the hospital during that year. These developments include increased complexity of patient care, shorter hospital stays, higher numbers of solid organ transplantations (SOT) and hematopoietic stem cell transplantations (HSCT), a greater number of patients with oncohematological conditions, and extended admission times for patients with cystic fibrosis. Our reported antimicrobial consumption rates for the initial year of the study (2015) were 70.6 DOT/100 PD, with the lowest consumption rates recorded in 2018 at 61.0 DOT/100 PD. Different studies conducted in highly complex third-level centers have analyzed antimicrobial consumption with varied results. In the US, children’s hospitals that implemented ASPs between 2007 and 2012 experienced a greater decrease in antibiotic use compared to those that did not, with a consumption rate of 69.3 DOT/100 PD after implementing ASPs [18]. A Canadian study reported stable antimicrobial consumption over a similar observational period, albeit at a substantially higher rate than ours (86.7 DOT/100 PD) [19]. A single-center study in the US reported a consumption of 78.7 DOT/100 PD [20], and a more recent study in the UK reported a consumption of 81.1 DOT/100 PD [21]. Another children’s hospital in our setting observed a reduction in antimicrobial consumption following the implementation of an ASP (68.4 DOT/100 PD pre-ASP vs. 65.6 DOT/100 PD post-ASP), although patients admitted to the P-ICU were excluded and the study lasted two years [36].

Comparing DOT/100 PD values across hospitals is challenging because of differences in information sources, data grouping methods, and access to specific antibiotics in each country, among others. To address these challenges, the WHO introduced the AWaRe classification as a global benchmarking tool [37,38]. Some experts have emphasized the urgent need to adopt consumption indicators based on the AWaRe classification to facilitate global interpretations and comparisons [39]. A study conducted in 56 countries worldwide highlighted substantial heterogeneity in the use of the different AWaRe groups among countries and centers. In Spain, Access antibiotics were used in 58.9% of children and more than 80% of neonates [38]. In our study, the Access antibiotic consumption rate (43.2%) was significantly lower than that reported in the multicenter study [38] and fell within the mid-range of values reported by other single-center studies [19,21]. However, there was significant variation in the consumption rates reported in different studies employing this classification, possibly due to differences in the complexity of care among centers, underlining the importance of standardization. Minimizing the use of antibiotics classified as Reserve is of relevance, particularly in the ICU. In our study, only 2.4% of patients received Reserve antibiotics, with minimal use in neonates, critical care, and surgical units.

The most commonly used antibiotics were combinations of penicillin with beta-lactamase inhibitors, consistent with earlier studies in tertiary pediatric hospitals [19,21,36]. However, a decrease in the consumption of antibiotics within this category was observed, probably due to a shift from using amoxicillin-clavulanic acid to gentamicin or cefuroxime for urinary tract infections. Notably, we observed a reduction in the consumption of glycopeptides during the initial three years of the program, primarily attributed to a decrease in the P-ICU, as previously observed after the implementation of an ASP [40].

The inappropriate use of antimicrobials can lead to adverse interactions, toxicity, increased mortality, and the emergence of resistant strains. Although data on pediatric antimicrobial use is limited, a multicenter study conducted in England found that only 34% of antifungal prescriptions were appropriate [41]. We recently published that 89% of prescriptions were appropriate after the introduction of the PROA-NEN program [42]. Likewise, one of the most striking findings of this present study was the significant improvement in prescription quality following the introduction of the PROA-NEN program. During the first two years, the mean rate of appropriate antibiotic prescription was 77.5%, which rose to 90% in the cross-sectional analyses conducted in 2018 and 2019. This improvement coincided with the increase in clinical counseling sessions (5046 sessions in 2016, 5114 in 2017, 5754 in 2018, and 5849 in 2019), highlighting the relevance of the program in enhancing prescription quality. Rates of appropriate prescription were also higher than those published in similar studies [36,43,44,45]. Our study also revealed an association between antibiotic treatment records and appropriate prescribing, confirming the importance of having comprehensive clinical records in improving the quality of antibiotic prescriptions. In a recent publication, we reported that the PROA-NEN program also reduced the rate of unnecessarily prolonged antibiotic treatments [46].

Resistance studies within the pediatric population in Spain are scarce [47,48]. Our research adds valuable data on the prevalence of antibiotic resistance and changes in resistance patterns of the most relevant bacteria from an epidemiological perspective. Such insights are pivotal for improving empirical prescribing practices. Our research also offers crucial tools for epidemiological surveillance, supporting ongoing infection control policies in healthcare settings, and provides benchmarks for comparisons with centers of similar size and complexity of care. Importantly, our results showed a decline in the prevalence of specific resistance mechanisms most directly linked to our antibiotic policy, such as *Enterobacter* spp. resistance to third-generation cephalosporins due to hyperproduction of chromosomal AmpCs, and carbapenem-resistant *Pseudomonas aeruginosa*.

This study had several strengths. It thoroughly evaluated antimicrobial consumption trends, prescription quality, clinical and microbiological outcomes, and costs of a pediatric ASP over an extended period. The inclusion of all pediatric patients admitted to the center, rather than focusing on a single area or department, provided a holistic view of the program’s impact. Additionally, the use of detailed cross-sectional analyses to assess prescription quality and the incorporation of extensive clinical counseling sessions underscored the program’s effectiveness in improving prescribing practices. Furthermore, the research offered valuable insights into antimicrobial resistance patterns within the pediatric population, thereby supporting ongoing infection control policies and providing benchmarks for future comparisons.

Our study presented some limitations worth noting. The use of antibiotic combinations may have been overestimated when compared to broad-spectrum compounds, given that each prescribed antibiotic was counted as a DOT. Additionally, due to resource constraints, we evaluated prescription quality in cross-sectional analyses rather than across the entire cohort. This study’s assessment of economic efficiency driven by the ASP only considered the impact of the centralized preparation of antimicrobials; however, a comprehensive pharmacoeconomic study of the ASP has not been conducted. Lastly, factors beyond the PROA-NEN program, such as the infection control program, may have influenced the evaluation of clinical and microbiological indicators, so these outcomes should be analyzed with caution.

This study was conducted in a single center and focused exclusively on hospital consumption. Our outcomes suggest that the PROA-NEN program and data collection from multicenter registries of antimicrobial consumption could be extended to other hospitals to generate a more in-depth analysis. We also believe a specific analysis of antimicrobial consumption by clinical entity would be useful. This represents a critical area for future exploration of the PROA-NEN program data in the coming years.

## 4. Methods

### 4.1. Intervention

The PROA-NEN was a non-restrictive (open to new interventions) program based on the available guidelines and recommendations [4,24] that was developed with the support of the hospital management and in coordination with the hospital’s infectious diseases board and antimicrobial subcommittee. A multidisciplinary team of 18 professionals, including a core team of three pediatric infectious diseases experts, a hospital pharmacist, and a microbiologist, led the program. Main tasks included training activities, protocol development, weekly or biweekly clinical counseling with feedback sessions across units using antimicrobials, and monitoring antimicrobial consumption, resistance and clinical indicators. Key tools of the PROA-NEN program consisted of real-time accessible computerized medical records and e-prescribing tools.

### 4.2. Design

A quasi-experimental, single-center study was conducted to evaluate the evolution of antimicrobial consumption, clinical and resistance indicators, and costs during the first five years after the introduction of the program. The quality of antibiotic prescription was also assessed by quarterly point prevalence cross-sectional analyses. This study protocol received approval from the Research Ethics Committee of the Children’s Hospital at Vall d’Hebron Barcelona Hospital Campus in May 2018 (PROA-NEN/HUV-ANT-2017-01).

### 4.3. Population

This study included pediatric patients (ages 0–18 years) admitted to the Children’s Hospital at Vall d’Hebron Barcelona Hospital Campus between January 2015 and December 2019. This included admissions to all pediatric medical and surgical specialty units, the P-ICU and the N-ICU, as well as patients receiving specialized pediatric care encompassing SOT, HSCT, cell therapies, oncohematological care, palliative care, and treatments for conditions like cystic fibrosis and primary immunodeficiencies.

Excluded from the study were patients treated in the pediatric emergency department but without admission, those in the major outpatient surgery unit, healthy newborns admitted postpartum with their mothers, and those in hospital-at-home, day hospital programs, or receiving hemodialysis.

The Vall d’Hebron is a tertiary university hospital with 194 pediatric beds distributed as follows: 109 in medical specialty units, 24 in surgical units, 16 in the P-ICU, and 45 in the N-ICU.

### 4.4. Variables

The impact of the PROA-NEN program was assessed using process (consumption trends and prescription quality) and outcome indicators (clinical and microbiological). Consumption trends were expressed as DOTs, which quantify the days of treatment a patient has received, regardless of the dosage and dosing schedule of administration. Moreover, days of therapy adjusted for each healthcare activity by the length of stay were represented as PD. This was calculated using the formula DOT × 100/PD, with data presented as the sum of cases. The quality of antibiotic use was assessed using the AWaRe classification [37]. This tool categorizes antimicrobials as open access (Access), controlled use (Watch), or reserved use in the absence of therapeutic alternatives (Reserve).

The study incorporated an evaluation of the prescription appropriateness score, which determined the appropriateness of prescriptions based on adherence to local protocols and international guidelines regarding dose, duration, and route of administration. This assessment was introduced after the study began and was carried out over 12 quarterly cross-sectional analyses between 2016 and 2019. We considered data from patients who received at least one systemic antibiotic between 9:00 a.m. and 12:00 p.m. on the designated cross-sectional analysis day. The rate of recording antibiotic prescriptions was determined in the last cross-sectional analyses. Only prescriptions detailing a therapeutic indication, either empirical or targeted, were included. Records were considered ‘complete’ when the name of the antibiotic, dose, route of administration, clinical indication, and expected duration of the treatment were specified. Records were considered ‘incomplete’ if any of these parameters were missing. Records were categorized as ‘no records’ if none of the above parameters were available.

The following clinical indicators were assessed: length of stay, annual readmission rates (number of readmitted patients/number of treated patients × 100), and infection-related mortality (annual infection-associated deaths/total deaths × 100). Annual readmission rates were evaluated on days 15, 16–30, and 30. The annual complexity of care was represented using the WM-DRG. In this process, the number of cases per DRG was multiplied by its relative weight (using Spanish weights for each year) and then divided by the total cases in that unit. We calculated the WM-DRG for all patients within a specific unit, group, or provider.

To monitor changes in antimicrobial resistance, we regularly tracked the microorganisms and resistance patterns as recommended by the national consensus on ASP in Spanish hospitals, using the reference cut-off points (established by the Clinical Laboratory Standard Institute [CLSI] and by the European Committee of Antimicrobial Susceptibility Testing [EUCAST]) to define the resistant bacteria [4]. These indicators, adapted for the pediatric population, reflect both the impact of antibiotic pressure and local epidemiological factors. Clinical categorization of resistance studies was based on the annual guidelines of the European Committee on Antimicrobial Susceptibility Testing (http://www.eucast.org. Accessed on 15 November 2021).

Financial costs were derived by multiplying the total annual units of each drug consumed with the annual updated unit costs from the hospital’s public procurement contracts. Costs associated with centralized preparation of systemic antimicrobials for intravenous administration in pharmacy departments were determined by multiplying the number of vials used with the unit cost. Indirect preparation costs were not considered as they could not be exclusively attributed to antimicrobial preparation. Costs of non-centralized preparation were calculated by assuming the use of one vial per prescribed dose. Savings were calculated by subtracting centralized from non-centralized preparation costs.

### 4.5. Sources of Information

Data on antimicrobial use, treatments, and costs for patients admitted to hospital and surgical facilities were extracted from the e-prescribing systems Silicon^®^ v11 (Grifols International, S.A., Sant Cugat del Vallès, Spain) and SAP^®^ Business Objects™ (Web Intelligence^®^, Los Angeles, CA, USA). For ICU patients, data were retrieved from the Centricity™ Critical Care 8.1 SP7 software (General Electric Company, Boston, MA, USA). Baseline patient characteristics, microbiological data, and therapeutic indications were obtained from electronic medical records using the SAP© program (NetWeaver 7.0 SPS37, CA, USA).

### 4.6. Statistical Analyses

A linear regression model was used to identify trends over time, with DOT × 100/PD as the response variable and the period as the explanatory variable. The percentage of variability was expressed as R-squared. The threshold for statistical significance was set at 5%. Mean annual changes were calculated using the compound annual growth rate (CAGR), recommended by the ECDC as a measure to visualize the average annual change described as a percentage of consumption relative to the start year of the study period [49]. A chi-squared test was performed to analyze the relationship between the appropriateness of antibiotic prescription and the period.

Statistical analyses were conducted using the SAS v9.4 software (SAS Institute Inc., Cary, NC, USA), Microsoft^®^ Office Excel^®^ 2007 (12.0.4518.1014) and R statistical software (R version 3.5.2©, 2015 The R Foundation for Statistical Computing, Vienna, Austria).

## 5. Conclusions

This study showed that the PROA-NEN program improved antimicrobial stewardship in a pediatric referral hospital, with a consequent reduction in direct expenditure over four years post-implementation. Overall antimicrobial consumption, expressed as DOT/100 PD, was lower than that reported in other international children’s hospitals of similar complexity. Notably, we found a decrease in antibiotic use in units that care for patients who are highly vulnerable to infection, such as the P-ICU, N-ICU, and surgical units. Beyond consumption metrics, the PROA-NEN program was also associated with more appropriate use of antibiotics and antifungal agents without compromising clinical and microbiological outcomes for patients.

## Figures and Tables

**Table 1:** Total antibiotic and antifungal consumption. Data are expressed as DOT/100 PD both throughout the entire study period and on an annual basis. Antimicrobials are categorized according to the ATC classification.

DOT/100PD	2015	2016	2017	2018	2019	Consumption Trend 2015–2019	CAGR (%)	*p*-Value
ANTIBACTERIALS								
J01CR—Combinations of penicillins, incl. beta-lactamase inhibitors	17.76	13.79	11.5	12.32	14.27	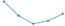	−4.3%	0.33
J01XA—Glycopeptide antibacterial agents	6.87	6.5	5.39	5.09	6.93	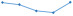	0.2%	0.7
J01CA—Penicillins with extended spectrum	5.72	5.69	6.26	5.6	6.26	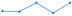	1.8%	0.41
J01DD—Third-generation cephalosporins	5.97	5.48	6.2	5.32	5.33	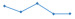	−2.2%	0.32
J01GB—Other aminoglycosides	4.81	4.83	4.75	4.57	5.63	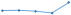	3.2%	0.35
J01DH—Carbapenems	5.19	5.08	3.04	3.74	5.18	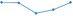	0.0%	0.73
J01FA—Macrolides	3.21	3.05	3.12	1.96	2.22	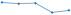	−7.1%	0.07
J01DC—Second-generation cephalosporins	1.39	1.6	2.23	2.3	2.68	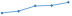	14.0%	0.01
J01MA—Fluoroquinolones	2.11	1.78	1.5	2.2	1.97	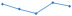	−1.4%	0.91
J01DB—First-generation cephalosporins	1.3	1.57	2.28	2	2.22	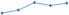	11.3%	0.07
J01CF—Beta-lactamase resistant penicillins	1.15	1	0.97	0.83	0.89	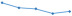	−5.0%	0.04
J01DE—Fourth-generation cephalosporins	0.64	0.7	1.41	0.92	1.04	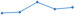	10.2%	0.36
J01EA—Trimethoprim and derivatives	1.03	0.61	0.88	1	1.11	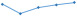	1.5%	0.45
J01XB—Polymyxins	0.67	0.83	0.75	0.63	1.6	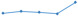	19.0%	0.23
J01CE—Beta-lactamase sensitive penicillins	0.76	0.58	0.79	0.89	0.92	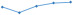	3.9%	0.14
J01XD—Imidazole derivatives	0.42	0.31	0.57	0.61	0.5	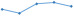	-	-
J01XX—Other antibacterials	0.33	0.11	0.24	0.36	0.31	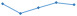	-	-
J01AA—Tetracyclines	-	0.02	0.08	0.1	0.4	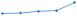	-	-
J01DF—Monobactams	0.14	0.14	0.04	0.16	0.08	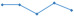	-	-
J01XE—Nitrofuran derivatives	0.01	0.06	-	-	-	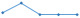	-	-
J01DI—Other cephalosporins and penems	-	-	-	0.06	-	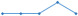	-	-
**ANTIFUNGALS**								
J02AC—Triazole and tetrazole derivatives	5.03	6.26	5.2	5.07	6.78	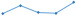	6.15%	0.44
J02AA—Antibiotics	5.06	4.71	3.53	4.27	4.51	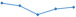	−2.28%	0.48
J02AX—Other antifungals for systemic use	1.06	0.85	0.58	0.95	1.02	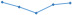	−0.77%	0.99

CAGR, compound annual growth rate.

**Table 2:** Changes in clinical indicators.

Indicator	2015	2016	2017	2018	2019	*p*-Value
LOS, days	7.98	8.29	7.42	7.62	7.22	0.10
Readmission rate at 15 days, %	5.43	5.51	5.47	5.44	5.54	0.38
Readmission rate at days 16–30, %	2.94	2.73	2.60	2.78	2.38	0.10
Readmission rate at day 30, %	8.37	8.25	8.07	8.22	7.92	0.07
Infection-related mortality, %	27.6	10.5	19.4	21.6	13.0	0.48

LOS, length of stay.

**Table 3:** Microbiological resistance indicators.

Resistance Indicator	Prevalence, % (Number of Resistant Cases/Number of Cases)				*p*-Value
	2016	2017	2018	2019	
*Escherichia coli* ESBL	9.2 (38/412)	9.5 (38/399)	8.3 (34/409)	8.2 (32/390)	0.16
*Klebsiella pneumoniae* ESBL	41.1 (30/73)	28.9 (28/97)	20.6 (20/97)	34.2 (41/120)	0.56
*Escherichia coli* FQR	17.7 (73/412)	22.3 (89/399)	21.3 (87/409)	20 (78/390)	0.61
*Escherichia coli* AMCR	30.8 (127/412)	28.3 (113/399)	31.3 (128/409)	49.7 (194/390)	0.22
Enterobacter 3GC-resistant (chrAmpC)	31.7 (13/41)	29.5 (13/44)	25 (11/44)	9.8 (5/51)	0.08
Enterobacteria CBP	1.5 (3/194)	0.3 (2/605)	0.3 (2/660)	1.4 (8/561)	0.94
*Pseudomonas aeruginosa* MR	24.8 (27/109)	18.1 (23/127)	12.6 (16/127)	16.1 (15/93)	0.20
*Pseudomonas aeruginosa* XDR	5.5 (6/109)	5.5 (7/127)	11.8 (15/127)	12.9 (12/93)	0.76
*Acinetobacter baumannii* MDR	0/16	0/24	0/12	0/6	-
VRE	0/40	0/58	0/64	0/68	-
MRSA	8.6 (17/197)	16.2 (38/235)	10.3 (30/290)	12.6 (30/237)	0.76

3GC, third-generation cephalosporins; AMCR, amoxicillin-clavulanic acid-resistant; CBP, carbapenemase; chrAmpC, chromosomal AmpC beta-lactamases; ESBL, extended-spectrum beta-lactamase; FQR, fluoroquinolone-resistant; MDR, multidrug-resistant; MR, meropenem-resistant; MRSA, methicillin-resistant *Staphylococcus aureus*; VRE, vancomycin-resistant enterococci; XDR, extremely resistant according to the criteria of Magiorakos et al. [17].

## Data Availability

The datasets that support the findings of this study are available from the corresponding author, AFP, upon request.

## Supplementary Materials

The following supporting information can be downloaded at https://www.mdpi.com/article/10.3390/antibiotics13060511/s1, Table S1: Summary of publications with antimicrobial consumption results [50].

## References

[1] Holmes A.H., Moore L.S.P., Sundsfjord A., Steinbakk M., Regmi S., Karkey A., Guerin P.J., Piddock L.J.V. (2016). Understanding the mechanisms and drivers of antimicrobial resistance. Lancet.

[2] World Health Organization (2015). Global Action Plan on Antimicrobial Resistance.

[3] Agencia Española de Medicamentos y Productos Sanitarios (2019). Plan Nacional Frente a la Resistencia a los Antibióticos 2019–2021. https://www.resistenciaantibioticos.es/es/publicaciones/plan-nacional-frente-la-resistencia-los-antibioticos-pran-2019-2021.

[4] Rodríguez-Baño J., Paño-Pardo J.R., Alvarez-Rocha L., Asensio Á., Calbo E., Cercenado E., Cisneros J.M., Cobo J., Delgado O., Garnacho-Montero J. (2012). Programas de optimización de uso de antimicrobianos (PROA) en hospitales españoles: Documento de consenso GEIH-SEIMC, SEFH y SEMPSPH. Farm. Hosp..

[5] de Salut D.G., Manual VINCat-PROA pediatria, Generalitat de Catalunya, Departament de Salut (2018). Dipòsit Legal: B 23446–2014.

[6] Gerber J.S., Jackson M.A., Tamma P.D., Zaoutis T.E., Maldonado Y.A., O’leary S.T., Banerjee R., Barnett E.D., Campbell J.D., Caserta M.T. (2021). Policy Statement: Antibiotic stewardship in pediatrics. J. Pediatric Infect. Dis. Soc..

[7] Dyar O.J., Huttner B., Schouten J., Pulcini C. (2017). What is antimicrobial stewardship?. Clin. Microbiol. Infect..

[8] Dyar O.J., Beović B., Pulcini C., Tacconelli E., Hulscher M., Barry Cookson D., Barcs I., Blix H.S., Buyle F., ESCMID generic competencies working group (2019). ESCMID generic competencies in antimicrobial prescribing and stewardship: Towards a European consensus. Clin. Microbiol. Infect..

[9] WHO, EMP, IAU (2018). WHO Global Framework for Development & Stewardship to Combat Antimicrobial Resistance Draft.

[10] Powell N., Davidson I., Yelling P., Collinson A., Pollard A., Johnson L., Gibson N., Taylor J., Wisner K., Gaze W. (2017). Developing a local antimicrobial resistance action plan: The Cornwall One Health Antimicrobial Resistance Group. J. Antimicrob. Chemother..

[11] Suwantarat N., Logan L.K., Carroll K.C., Bonomo R.A., Simner P.J., Rudin S.D., Milstone A.M., Tekle T., Ross T., Tamma P.D. (2016). The Prevalence and Molecular Epidemiology of Multidrug-Resistant Enterobacteriaceae Colonization in a Pediatric Intensive Care Unit. Infect. Control Hosp. Epidemiol..

[12] Fanelli U., Chiné V., Pappalardo M., Gismondi P., Esposito S. (2020). Improving the Quality of Hospital Antibiotic Use: Impact on Multidrug-Resistant Bacterial Infections in Children. Front. Pharmacol..

[13] Tsai M.-H., Chu S.-M., Hsu J.-F., Lien R., Huang H.-R., Chiang M.-C., Fu R.-H., Lee C.-W., Huang Y.-C. (2014). Risk factors and outcomes for multidrug-resistant Gram-negative bacteremia in the NICU. Pediatrics.

[14] Kosecka-Strojek M., Sadowy E., Gawryszewska I., Klepacka J., Tomasik T., Michalik M., Hryniewicz W., Miedzobrodzki J. (2020). Emergence of linezolid-resistant *Staphylococcus epidermidis* in the tertiary children’s hospital in Cracow, Poland. Eur. J. Clin. Microbiol. Infect. Dis..

[15] Basmaci R., Bielicki J., Daniels R., Kissoon N., Ellis S., Balasegaram M., Sharland M. (2018). Management of children with multidrug-resistant sepsis in low-income and middle-income countries. Lancet. Child Adolesc. Health.

[16] Bielicki J.A., Lundin R., Sharland M. (2015). Antibiotic resistance prevalence in routine bloodstream isolates from children’s hospitals varies substantially from adult surveillance data in Europe. Pediatr. Infect. Dis. J..

[17] Magiorakos A.P., Srinivasan A., Carey R.B., Carmeli Y., Falagas M.E., Giske C.G., Harbarth S., Hindler J.F., Kahlmeter G., Olsson-Liljequist B. (2012). Multidrug-resistant, extensively drug-resistant and pandrug-resistant bacteria: An international expert proposal for interim standard definitions for acquired resistance. Clin. Microbiol. Infect..

[18] Hersh A.L., De Lurgio S.A., Thurm C., Lee B.R., Weissman S.J., Courter J.D., Brogan T.V., Shah S.S., Kronman M.P., Gerber J.S. (2015). Antimicrobial stewardship programs in freestanding children’s hospitals. Pediatrics.

[19] Rahem L.R., Franck B., Roy H., Lebel D., Ovetchkine P., Bussières J.-F. (2021). Profile of Antimicrobial Use in the Pediatric Population of a University Hospital Centre, 2015/16 to 2018/19. Can. J. Hosp. Pharm..

[20] Newland J.G., Stach L.M., De Lurgio S.A., Hedican E., Yu D., Herigon J.C., Prasad P.A., Jackson M.A., Myers A.L., Zaoutis T.E. (2012). Impact of a prospective-audit-with-feedback antimicrobial stewardship program at a children’s hospital. J. Pediatr. Infect. Dis. Soc..

[21] Channon-Wells S., Kwok M., Booth J., Bamford A., Konstanty P., Hatcher J., Dixon G., Diggle P.J., Standing J.F., Irwin A.D. (2021). The use of continuous electronic prescribing data to infer trends in antimicrobial consumption and estimate the impact of stewardship interventions in hospitalized children. J. Antimicrob. Chemother..

[22] Gerber J.S., Jackson M.A., Tamma P.D., Zaoutis T.E. (2021). Antibiotic Stewardship in Pediatrics. Pediatrics.

[23] Probst V., Islamovic F., Mirza A. (2021). Antimicrobial stewardship program in pediatric medicine. Pediatr. Investig..

[24] Fishman N., Society for Healthcare Epidemiology of America and Infectious Diseases Society of America (2012). Policy Statement on Antimicrobial Stewardship by the Society for Healthcare Epidemiology of America (SHEA), the Infectious Diseases Society of America (IDSA), and the Pediatric Infectious Diseases Society (PIDS). Infect. Control Hosp. Epidemiol..

[25] Tersigni C., Venturini E., Montagnani C., Chiappini E., de Martino M., Galli L. (2019). Antimicrobial stewardship in children: More shadows than lights?. Expert Rev. Anti. Infect. Ther..

[26] Brett A., Bielicki J., Newland J.G., Rodrigues F., Schaad U.B., Sharland M. (2013). Neonatal and Pediatric Antimicrobial Stewardship Programs in Europe—Defining the Research Agenda. Pediatr. Infect. Dis. J..

[27] Godbout E.J., Pakyz A.L., Markley J.D., Noda A.J., Stevens M.P. (2018). Pediatric Antimicrobial Stewardship: State of the Art. Curr. Infect. Dis. Rep..

[28] Principi N., Esposito S. (2016). Antimicrobial stewardship in paediatrics. BMC Infect. Dis..

[29] Barlam T.F., Cosgrove S.E., Abbo L.M., Macdougall C., Schuetz A.N., Septimus E.J., Srinivasan A., Dellit T.H., Falck-Ytter Y.T., Fishman N.O. (2016). Implementing an antibiotic stewardship program: Guidelines by the Infectious Diseases Society of America and the Society for Healthcare Epidemiology of America. Clin. Infect. Dis..

[30] Newland J.G., Hersh A.L. (2010). Purpose and design of antimicrobial stewardship programs in pediatrics. Pediatr. Infect. Dis. J..

[31] McPherson C., Lee B.R., Terrill C., Hersh A.L., Gerber J.S., Kronman M.P., Newland J.G. (2018). Characteristics of pediatric antimicrobial stewardship programs: Current status of the sharing antimicrobial reports for pediatric stewardship (SHARPS) collaborative. Antibiotics.

[32] Guarch-Ibáñez B., Fernández-Polo A., Hernández S., Velasco-Arnaiz E., Giménez M., Sala-Castellvi P., Pineda V., Melendo S. (2023). VINCat Pediatric Proa Group. Assessment of the Plans to Optimize Antimicrobial Use in the Pediatric Population in Catalan Hospitals: The VINCat Pediatric PROA SHARP Survey. Antibiotics.

[33] Cercenado E., Rodríguez-Baño J., Alfonso J.L., Calbo E., Escosa L., Fernández-Polo A., García-Rodríguez J., Garnacho J., Gil-Navarro M.V., Grau S. (2022). Antimicrobial stewardship in hospitals: Expert recommendation guidance document for activities in specific populations, syndromes and other aspects (PROA-2) from SEIMC, SEFH, SEMPSPGS, SEMICYUC and SEIP. Enferm. Infecc. Microbiol. Clin..

[34] Sweileh W.M. (2021). Bibliometric analysis of peer-reviewed literature on antimicrobial stewardship from 1990 to 2019. Global. Health.

[35] Donà D., Barbieri E., Daverio M., Lundin R., Giaquinto C., Zaoutis T., Sharland M. (2020). Implementation and impact of pediatric antimicrobial stewardship programs: A systematic scoping review. Antimicrob. Resist. Infect. Control.

[36] Velasco-Arnaiz E., Simó-Nebot S., Ríos-Barnés M., López Ramos M.G., Monsonís M., Urrea-Ayala M., Jordan I., Mas-Comas A., Casadevall-Llandrich R., Ormazábal-Kirchner D. (2020). Benefits of a Pediatric Antimicrobial Stewardship Program in Antimicrobial Use and Quality of Prescriptions in a Referral Children’s Hospital. J. Pediatr..

[37] World Health Organization (2019). The 2019 WHO AWaRe Classification of Antibiotics for Evaluation and Monitoring of Use.

[38] Hsia Y., Lee B.R., Versporten A., Yang Y., Bielicki J., Jackson C., Newland J., Goossens H., Magrini N., Sharland M. (2019). Use of the WHO Access, Watch, and Reserve classification to define patterns of hospital antibiotic use (AWaRe): An analysis of paediatric survey data from 56 countries. Lancet Glob. Health.

[39] Donà D., Sharland M. (2021). The Urgent Need for Simple and Globally Applicable Quality Indicators of Optimal Prescribing for Children Using the Access, Watch, Reserve (AWaRe) System. J. Pediatr. Infect. Dis. Soc..

[40] Renk H., Sarmisak E., Spott C., Kumpf M., Hofbeck M., Hölzl F. (2020). Antibiotic stewardship in the PICU: Impact of ward rounds led by paediatric infectious diseases specialists on antibiotic consumption. Sci. Rep..

[41] Ferreras-Antolín L., Irwin A., Atra A., Dermirjian A., Drysdale S.B., Emonts M., McMaster P., Paulus S., Patel S., Kinsey S. (2019). Neonatal Antifungal Consumption Is Dominated by Prophylactic Use; Outcomes From The Pediatric Antifungal Stewardship. Pediatr. Infect. Dis. J..

[42] Mendoza-Palomar N., Garcia-Palop B., Melendo S., Martín M.T., Renedo-Miró B., Soler-Palacin P., Fernández-Polo A. (2021). Antifungal stewardship in a tertiary care paediatric hospital: The PROAFUNGI study. BMC Infect. Dis..

[43] Osowicki J., Gwee A., Noronha J., Palasanthiran P., McMullan B., Britton P.N., Isaacs D., Lai T., Nourse C., Avent M. (2014). Australia-wide point prevalence survey of the use and appropriateness of antimicrobial prescribing for children in hospital. Med. J. Aust..

[44] Gharbi M., Doerholt K., Vergnano S., Bielicki J.A., Paulus S., Menson E., Riordan A., Lyall H., Patel S.V., Bernatoniene J. (2016). Using a simple point-prevalence survey to define appropriate antibiotic prescribing in hospitalised children across the UK. BMJ Open.

[45] McMullan B.J., Hall L., James R., Mostaghim M., Jones C.A., Konecny P., Blyth C.C., Thursky K.A. (2020). Antibiotic appropriateness and guideline adherence in hospitalized children: Results of a nationwide study. J. Antimicrob. Chemother..

[46] Melendo S., Fernández-Polo A., Castellnou Asens I., Mendoza-Palomar N., Barnés-Mayolas M., Soler-Palacín P., Soler Palacín P., Melendo Pérez S., Mendoza Palomar N., Fernández Polo A. (2021). Prescription quality of prolonged antibiotherapy in pediatrics. Impact of ASP program interventions. Enfermedades Infecc. Microbiol. Clin. (Engl. Ed.).

[47] Ara-Montojo M.F., Escosa-García L., Alguacil-Guillén M., Seara N., Zozaya C., Plaza D., Schuffelmann-Gutiérrez C., de la Vega Á., Fernández-Camblor C., Ramos-Boluda E. (2021). Predictors of mortality and clinical characteristics among carbapenem-resistant or carbapenemase-producing Enterobacteriaceae bloodstream infections in Spanish children. J. Antimicrob. Chemother..

[48] Gimenez M., Monsonis M., Larrosa N., Perez P., Rivera A., Gómez F., Bernet A., Trujillo G., Clapes E., Llaberia J. (2020). First paediatric antimicrobial resistance surveillance network, including community and healthcare settings, carried out by 13 hospitals in Catalonia (Spain). Proceedings of the European Congress of Clinical Microbiology and Infectious Diseases.

[49] ECDC (2020). Antimicrobial Consumption in the EU/EEA—Annual Epidemiological Report 2019. https://www.ecdc.europa.eu/en/publications-data/surveillance-antimicrobial-consumption-europe-2020.

[50] Stultz J.S., Kohinke R., Pakyz A.L. (2018). Variability in antifungal utilization among neonatal, pediatric, and adult inpatients in academic medical centers throughout the United States of America. BMC Infect. Dis..

