# Supplementary material for: Five-Year Evaluation of the PROA-NEN Pediatric Antimicrobial Stewardship Program in a Spanish Tertiary Hospital
Source: Antibiotics (Basel). 2024 May 30;13(6):511. doi: 10.3390/antibiotics13060511 (PMC11200588; doi:10.3390/antibiotics13060511)
Supplement: Supplementary file 1 [file antibiotics-13-00511-s001.zip › antibiotics-3008090-supplementary.pdf]

Supplementary material Table S1. Summary of publications with antimicrobial consumption results

STUDIES	TYPE OF STUDY	CENTER CHARACTERISTICS	ANTIMICROBIAL CONSUMPTION RESULTS
Newland et *al.,* 2012. Children's Mercy Hospitals & Clinics. (United States) [20]	Single-center. Quasi-experimental study with control group.	Third-level children's hospital with 317 beds. Clinical units: N-ICU, P-ICU, OH, complex congenital heart diseases, SOT, and HSCT.	**Antibiotics:** 88.3 DOT/100 PD Pre-ASP implementation vs. 78.7 dot/100PD post-ASP ($p < 0.001$)
Velasco-Armaiz et *al*., 2020. Hospital Sant Joan de Déu. Barcelona, (Catalonia, Spain) [36]	Single-center. Interrupted time series study. Cross-sectional prevalence.	Third-level children's hospital, 268 beds. Medical and surgical subspecialties.	**Antimicrobials**: 68.4 DOT/100 days present pre-ASP implementation vs. 65.6 DOT/100 days present post-ASP ($p <$ 0.044)<br>· Antibiotics: 63.4 DOT/100 days present pre-ASP implementation vs. 57.5 DOT/100 days present post-ASP ($p < 0.001$)<br>· Antifungals 4.9 DOT/100 days present pre-ASP implementation vs. 6.6 DOT/100 days present post-ASP ($p < 0.001$)
Rahem et *al.,* 2021. CHU-Sainte Justine. Montreal (Canada) [19]	Single-center. Retrospective cross-sectional study.	Third-level children's hospital, 500 beds. Clinical units: surgery, neonatology, OH, pediatrics, psychiatry, rehabilitation, and P-ICU	**Antimicrobials**: 88.6 DOT/100 PD pre-ASP implementation vs. 86.7DOT/100PD post-ASP<br>· Antibiotics: 75.6 DOT/100 PD pre-ASP implementation vs. 73.1 DOT/100 PD<br>· Antifungals: 8.8 DOT/100 PD pre-ASP implementation vs. 8.0 DOT/100 PD post-ASP vs.
Channon et *al.,* 2021. Great Ormond Street Hospital. London (UK) [21]	Single-center. Retrospective study,	Third-level children's hospital. Clinical units: Pediatric medical and surgical specialties, neonates, cardiology, P-ICU, SOT, OH and HSCT.	**Antimicrobials**: 84.4 DOT/100 PD pre-ASP implementation vs 81.1 DOT/100 PD post-ASP<br>· Antibiotics in non-P-ICU patients: 61.7 DOT/100 PD<br>· Antibiotics in P-ICU patients: 141.1 DOT/100 PD

Hersh et *al.,* 2015. (United States) [18]	Multicenter. Interrupted time series study.	31 children's hospitals in the United States.	**Antibiotics in hospitals with ASP**: 77.5 DOT/100 PD pre-ASP implementation vs. 69.3 DOT/100 PD<br>Antibiotics in hospitals without ASP: 77.1 DOT/100 PD vs. 70.9 DOT/100 PD
Stultz et *al.,* 2018. (United States) [50]	Multicenter. Cross-sectional study.	54 hospitals with neonatal departments and 44 pediatric hospitals. Hospitals with only oncology care were excluded.	**Antifungals in neonates:** 1.4 DOT/100 PD<br>Antifungals in pediatrics: 7.6 DOT/100 PD
VINCat Pediatric Report, 2021. Barcelona (Catalonia, Spain) [5]	Multicenter. Cross-sectional study.	18 hospitals with pediatric departments.	**Antibiotics:** 53.5 DOT/100 PD<br>· Level 1 hospitals: 50.8 DOT/100 PD<br>· Level 2 hospitals: 44.9 DOT/100 PD<br>· Level 3 hospitals: 56.0 DOT/100 PD
Fernandez-Polo et *al.* Barcelona, (Catalonia, Spain) (actual data)	Single-center. Interventional, quasi-experimental, interrupted time series study.	Third-level children's hospital, 194 beds. Pediatric clinical units: pediatric medical and surgical specialties, neonates, P-ICU, N-ICU, SOT, OH, HSCT and CF, complex congenital heart disease and immunodeficiencies.	**Antimicrobials**: 70.6 DOT/100 PD pre-ASP implementation vs. 71.9 dot/100 PD post-ASP ($p < 0.004$)<br>· Antibiotics: 59.5 DOT/100 PD pre-ASP implementation vs. 59.6 dot/100 PD post-ASP ($p < 0.011$)<br>· Antifungals: 11.1 DOT/100 PD pre-ASP implementation vs. 12.3 dot/100PD post-ASP ($p < 0.011$)

DOT, days of therapy; CF, cystic fibrosis; HSCT, hematopoietic stem transplantation; N-ICU, neonatal intensive care unit; OH, oncologyhematology; PD, patient days; P-ICU, pediatric intensive care unit; SOT, solid organ transplantation
